# Antiviral activity of *Lactobacillus reuteri* Protectis against Coxsackievirus A and Enterovirus 71 infection in human skeletal muscle and colon cell lines

**DOI:** 10.1186/s12985-016-0567-6

**Published:** 2016-06-24

**Authors:** Lei Yin Emily Ang, Horng Khit Issac Too, Eng Lee Tan, Tak-Kwong Vincent Chow, Pei-Chi Lynette Shek, Elizabeth Tham, Sylvie Alonso

**Affiliations:** Department of Microbiology and Immunology, Yong Loo Lin School of Medicine, National University of Singapore, Centre for Life Sciences, 28 Medical Drive, #03-05, Singapore, 117456 Singapore; Immunology programme, Life Sciences Institute, National University of Singapore, Singapore, Singapore; Department of Paediatrics, National University Hospital, Singapore, Singapore; Centre for Biomedical & Life Sciences, Singapore Polytechnic, Singapore, Singapore

**Keywords:** Hand, Foot and mouth disease, Probiotics, *Lactobacillus reuteri*, Coxsackievirus, Enterovirus 71

## Abstract

**Background:**

Recurrence of hand, foot and mouth disease (HFMD) pandemics continues to threaten public health. Despite increasing awareness and efforts, effective vaccine and drug treatment have yet to be available. Probiotics have gained recognition in the field of healthcare worldwide, and have been extensively prescribed to babies and young children to relieve gastrointestinal (GI) disturbances and diseases, associated or not with microbial infections. Since the faecal-oral axis represents the major route of HFMD transmission, transient persistence of probiotic bacteria in the GI tract may confer some protection against HFMD and limit transmission among children.

**Methods:**

In this work, the antiviral activity of two commercially available probiotics, namely *Lactobacillus reuteri* Protectis (*L. reuteri* Protectis) and *Lactobacillus casei* Shirota (*L. casei* Shirota), was assayed against Coxsackieviruses and Enterovirus 71 (EV71), the main agents responsible for HFMD. In vitro infection set-ups using human skeletal muscle and colon cell lines were designed to assess the antiviral effect of the probiotic bacteria during entry and post-entry steps of the infection cycle.

**Results:**

Our findings indicate that *L. reuteri* Protectis displays a significant dose-dependent antiviral activity against Coxsackievirus type A (CA) strain 6 (CA6), CA16 and EV71, but not against Coxsackievirus type B strain 2. Our data support that the antiviral effect is likely achieved through direct physical interaction between bacteria and virus particles, which impairs virus entry into its mammalian host cell. In contrast, no significant antiviral effect was observed with *L. casei* Shirota.

**Conclusions:**

Should the antiviral activity of *L. reuteri* Protectis observed in vitro be translated in vivo, such probiotics-based therapeutic approach may have the potential to address the urgent need for a safe and effective means to protect against HFMD and limit its transmission among children.

## Background

Hand, foot and mouth disease (HFMD) is a common viral infection that affects mostly infants and children below 5 years of age. The main causative agents responsible for HFMD belong to a group of enteroviruses from *Picornaviridae* family, and consist predominantly of coxsackievirus type A (CA) strain 16 (CA16) and enterovirus 71 (EV71) [1]. Other enteroviruses such as CA6, CA7, CA10, CA14 and coxsackievirus type B strain 2 (CB2) may also associate with the disease. In most cases, the disease is mild and self-limiting, with major clinical features manifesting as HFMD and herpangina [2, 3]. However, more severe clinical manifestations with neurological complications including aseptic meningitis, brainstem encephalitis, acute flaccid paralysis and cardiopulmonary dysfunction resulting from acute EV71 infection, have also been reported [3, 4]. Furthermore, co-infection with CA16 and EV71 has been detected in patients [5]. A growing body of evidence suggests that overwhelming production of inflammatory mediators associated with high viral titer plays a critical role in the pathogenesis of EV71 infection [3, 6, 7].

In the past decade, epidemiology studies of HFMD outbreaks resulting in morbidity and mortality with neurological complications have been increasingly reported in countries across the Asia-Pacific region and sometimes in Europe [8–11]. However, there is still no effective vaccine and specific antiviral treatment available currently. Infection risk control is mainly achieved through good hygiene practices, closure of childcare centres and schools, and adopting distancing measures. However, these measures imply a substantial socio-economic burden [7]. Efforts in developing suitable vaccines have been pursued to address the urgent need to control HFMD epidemics [12, 13]. So far three inactivated EV71 whole-virus vaccine candidates have completed Phase III clinical trials. These C4 genotype-based vaccines showed high immunogenicity and good protective efficacy by preventing herpangina and EV71-associated hospitalization. In addition, they were shown to cross-neutralize the circulating EV71 predominant genotypes and subgenotypes B1, B5 and C4A which have been associated with epidemics in recent years. However, no cross-protection against CA16 was observed [14, 15].

Probiotics, as defined by the Food and Agricultural Organization of the United Nations and World Health Organization, are “live microorganisms which, when administered in adequate amounts, confer a health benefit on the host” [16]. Lactic acid bacteria (LAB) and bifidobacteria are the most common types of probiotics. They are widely consumed as part of fermented foods with specially added active live cultures; such as in yogurt, soy yogurt, or as dietary supplements. Probiotics were initially thought to exert a beneficial effect on the host by improving intestinal microbial balance, through inhibition of, or competition with pathogens and toxin-producing bacteria. It was later shown that probiotics seem to display more specific health effects that are being increasingly investigated and documented [17]. An extensive scientific literature is available on the effects of probiotics in alleviating chronic intestinal inflammatory diseases [18], preventing and treating pathogen- or antibiotic-induced diarrhoea [19], urogenital infections [20], and atopic diseases [21]. Immuno-modulatory activities were reported for some LAB strains through the regulation of cytokine production, by increasing the number of IgA-producing plasma cells or the proportion of T lymphocytes and Natural Killer cells, or by improving phagocytosis [22, 23]. Clinical trials have further demonstrated that probiotics may decrease the incidence of respiratory tract infections [24] and dental caries in children [25].

Since the faecal-oral axis represents the major route of HFMD transmission [7], transient persistence of probiotic bacteria in the gastrointestinal (GI) tract may confer some protection against HFMD and limit transmission among children. Consistently, a previous publication has reported the anti-EV71 activity of metabolites secreted by *Lactobacillus plantarum* and *Bifidobacterium bifidum* in Vero cells [26]. Here, we studied the potential antiviral activity of two commercially available LAB probiotic, namely *Lactobacillus reuteri* Protectis (*L. reuteri* Protectis) and *Lactobacillus casei* Shirota (*L. casei* Shirota), against coxsackieviruses type A and B, and EV71 in infection assays using human skeletal muscle and colon cells. *L. reuteri* Protectis, commercialized by BioGaia, was shown to improve gut health in infants and children [27–30]. *L. casei* Shirota contained in Yakult products has also been demonstrated to relieve gastrointestinal symptoms, prevent viral infections and reduce risk for various cancers [31–35]. Most importantly, both probiotics are safe to consume in clinically ill children.

## Methods

### Bacteria strains and growth conditions

*L. reuteri* Protectis (Deutsche Sammlung von Mikroorganismen 17938) [36] was re-activated from freeze-dried BioGaia ProTectis tablet. *L. casei* Shirota is a kind gift from A/Prof Lee Yuan Kun (Department of Microbiology and Immunology, National University of Singapore). Both *L. reuteri* Protectis and *L. casei* Shirota were grown in MRS broth or on MRS agar (Oxoid, United Kingdom) at 37 °C. All LAB cultures were incubated under microaerobic condition (closed cap without agitation) to stationary phase, between 16 and 18 h, to achieve a bacterial concentration of 10^12^ colony-forming unit per mL (CFU/mL). Bacteria cultures were passaged twice from frozen stock ( −80 °C). Formaldehyde-inactivated *L. reuteri* Protectis was obtained by resuspending live bacteria pellet in phosphate buffered saline (PBS) containing 4 % v/v formaldehyde (Sigma-Aldrich, United States) overnight. Prior to infection assays bacteria were washed extensively with PBS to remove traces of MRS broth or formaldehyde, and serially diluted in Dulbecco’s Modified Eagle Medium (DMEM) supplemented with 2 % (v/v) heat-inactivated fetal bovine serum (FBS).

### Cultures of cell lines and virus strains

Human rhabdomyosarcoma (RD) cells (ATCC® CCL-136™) were maintained in DMEM supplemented with 10 % v/v heat-inactivated FBS. Human Caco-2 cells (ATCC® HTB-37™) were cultured in DMEM containing 20 % v/v heat-inactivated FBS, 1 % v/v non-essential amino acids (100X), 1 % v/v GlutaMAX supplement (100X), 1.5 % v/v sodium bicarbonate (7.5 %) and 1 % v/v sodium pyruvate (100 mM). Both cell lines were culture in 5 % CO_2_ atm at 37 °C, and were sub-cultured every 2–3 days. RD cells and Caco-2 cells were used between passages 13 and 40. All the virus strains, CA6 (NUH0026/SIN/08, Accession No. GU198758.1), CA16 (CA16-G-10, Accession No. U05876) [37], CB2 (KOR 04-279, Accession No. EF174469) and EV71 strain 41 (5865/SIN/00009, Accession No. AF316321) [38], used for this study were propagated in RD cells. All the reagents used to maintain cell cultures were purchased from Thermo Fisher Scientific (Gibco).

### Immunostaining assay

*L. reuteri* Protectis bacteria (10^11^ CFU) were co-incubated with CA16 (10^5^ PFU) in 2 % DMEM at 37 °C for one hour. The mixture was then added to RD cells (10^5^ cells) for another hour to allow viral entry. CA16-infected RD cells served as control. After one hour incubation, the cells were washed thrice with PBS to remove unbound bacteria and viruses, and immediately fixed with ice cold methanol. The cells were then stained with mouse anti-CA16 antibody (MAB979, Merck, 1:1000 dilution) and rabbit anti-beta actin antibody (ab8227, Abcam, 1:1,000 dilution) followed by incubation with anti-mouse AF488-conjugated (A-11001, Invitrogen, 1:500 dilution) and anti-rabbit AF594-conjugated (R37117, Invitrogen, 1:500 dilution) secondary antibodies, respectively. Cell nuclei were stained with 4',6’-diamidino-2-phenylindole (DAPI) (Invitrogen) (1:100,000 dilution) at room temperature for 30 min in the dark. Images were captured using Olympus IX81 microscope. Cell fluorescence (viral signals, green) was measured using ImageJ software and the corrected total cell fluorescence (CTCF) was calculated using the equation: CTCF = Raw integrated Density – (Area of selected cell × Mean fluorescence of background readings).

### Cell viability assay

RD cells (2.5 × 10^4^ cells/well) and Caco–2 cells (5 × 10^3^ cells/well) were seeded onto 96 well plates (Nunc, United States) and incubated overnight and for 6 days, respectively. The culture medium was changed every 2- 3 days. Monolayers were incubated with various concentrations of live bacteria for an hour and washed thrice with PBS before fresh 2 % DMEM supplemented with 50 μg/mL gentamicin (Sigma-Aldrich, United States) was added. After 24 h incubation at 37 °C and CO_2_, 2 % v/v alamarBlue reagent (Invitrogen, United States) diluted in 2 % DMEM was added to each well. Fluorescence intensity was then measured at excitation wavelength of 570 nm and emission wavelength of 585 nm using a microplate reader (Infinite 200, Tecan). The relative percentage (%) of cell survival with respect to control wells containing untreated cells was calculated. Values were corrected for background fluorescence obtained with media only.

### Virus quantification

RD cells (1.25x10^5^ cells/well) were seeded onto 24 well plates (Nunc) and infected with 200 μL of 10-fold serially diluted viral supernatant. After 1 h incubation at 37^0^C and CO_2_, 1 % w/v sodium carboxymethyl cellulose (Sigma-Aldrich) in Minimum Essential Medium (MEM) (Invitrogen, United States) supplemented with 2 % v/v heat-inactivated FBS and 1.5 % v/v sodium bicarbonate (7.5 %) was added to the wells. After 3 days incubation, the cells were fixed with 4 % v/v formaldehyde and stained with 1 % w/v crystal violet (Sigma-Aldrich). Plaques were scored visually and the virus titers were expressed as plaque-forming units per mL (PFU/mL). Three technical replicates were performed for each dilution of a biological sample. The limit of detection for the plaque assay was set at 10 PFU/mL.

### Antiviral activity assays

Four experimental set-ups were designed namely, pre-incubation, pre-treatment, co-treatment and post-treatment. Virus infection was carried out at a multiplicity of infection (MOI) of 1 (10^5^ PFU/mL) for all the set-ups. Cell monolayers were washed with PBS thrice and were maintained in 2 % DMEM supplemented with 50 μg/mL of gentamicin after contact time with bacteria. Culture supernatant was harvested at 12 (CB2-infection) or 24 (CA6, CA16, or EV71-infection) hours post-infection. Samples were stored in −80 °C and plaque assay was performed subsequently to determine the viral titer. The virus titers were compared with the titer obtained with cells exposed to virus only.

### Virus-bacteria binding assay

Live *L. reuteri* Protectis bacteria were co-incubated with CA6, CA16, EV71 or CB2 virus in 2 % DMEM at 37 °C and CO_2_ for an hour. The bacteria-virus mixtures were then spun down at 4,000 *x g* for 5 min at 4 °C. The culture supernatant was filtered with 0.22 μm syringe filter unit (Millipore, United States) before viral titer determination in RD cells (section 2.4).

### Statistical analysis

The results were expressed as mean ± standard deviation (SD) of three technical replicates. All the experiments were performed twice independently. Comparison between control and different treatment groups was statistically analyzed by one-way analysis of variance (ANOVA) with Dunnett’s post-test or by Mann–Whitney *U* test as indicated, using GraphPad Prism version 5.00 for Windows, GraphPad Software (San Diego California USA, www.graphpad.com). Probability values (*p*) of < 0.05 were considered statistically significant.

## Results

### Live *L. reuteri* Protectis bacteria significantly reduced CA6, CA16, EV71 but not CB2 virus titers in infected RD and Caco-2 cells

**Fig. 1:**
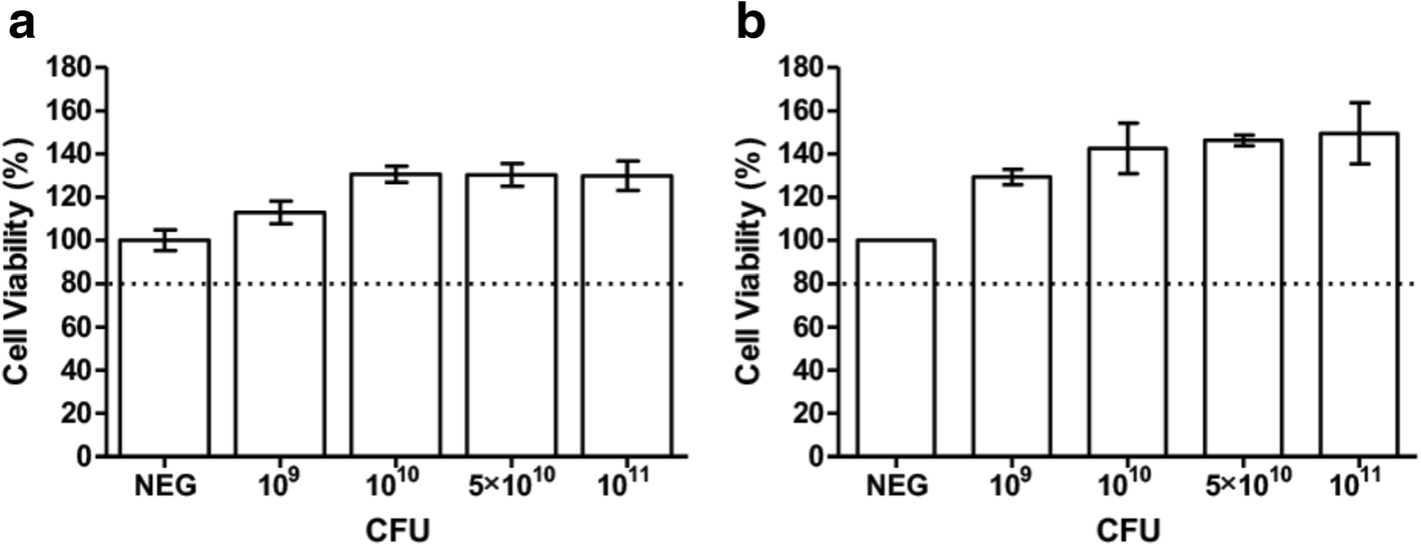
Cell viability in the presence of live *L. reuteri* Protectis bacteria. Different concentrations of *L. reuteri* Protectis bacteria were added to RD cells (**a**) and Caco-2 cells (**b**) and incubated for 1 h, then washed with PBS thrice before 50 μg/ml gentamicin-supplemented maintenance media was added to the cells. Alamar blue assay was performed at 24 h post-treatment according to the manufacturer’s instructions. Data are expressed as the mean ± standard deviation of technical triplicates. Two biological repeats were conducted. One representative is shown

**Fig. 2:**
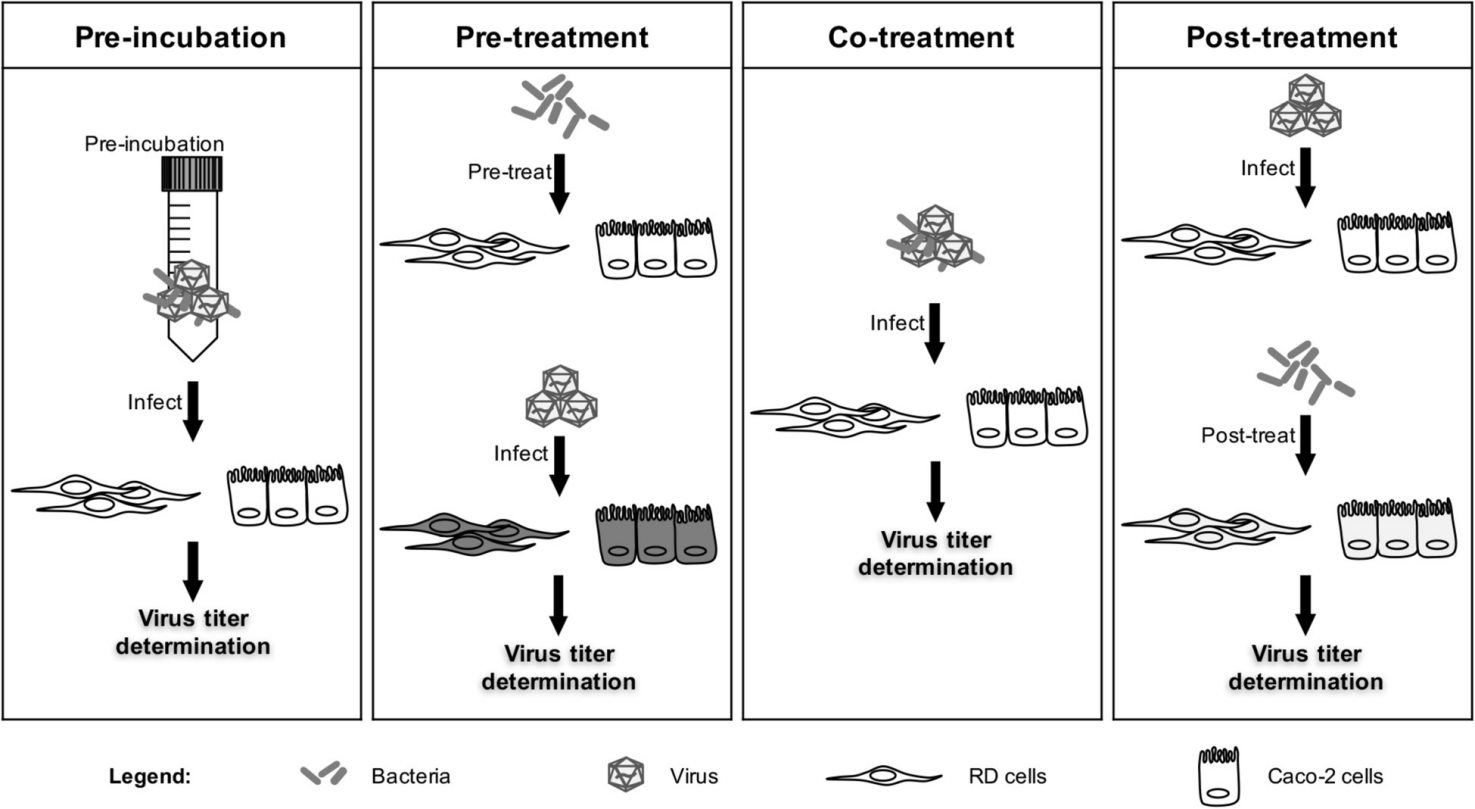
Schematic diagram of experimental *in vitro* set-ups. In the pre-incubation set-up, live bacteria and virus were pre-incubated for 1 h at 37 °C, before being incubated with the mammalian cells for 1 h. In the pre-treatment set-up, the mammalian cells were incubated with live bacteria for 1 h, washed with PBS and infected with virus for 1 h. In the co-treatment set-up, live bacteria were added to the cells at the same time of virus infection for 1 h. In the post-treatment set-up, the mammalian cells were infected with virus for 1 h, then washed with PBS and incubated with live bacteria for another hour. In all four set-ups, 50 μg/ml gentamicin-supplemented maintenance media was eventually added to the cell monolayers. Sample supernatants were harvested 12 or 24 h post-infection and plaque assay was performed using RD cells to determine the viral titer

**Fig. 3:**
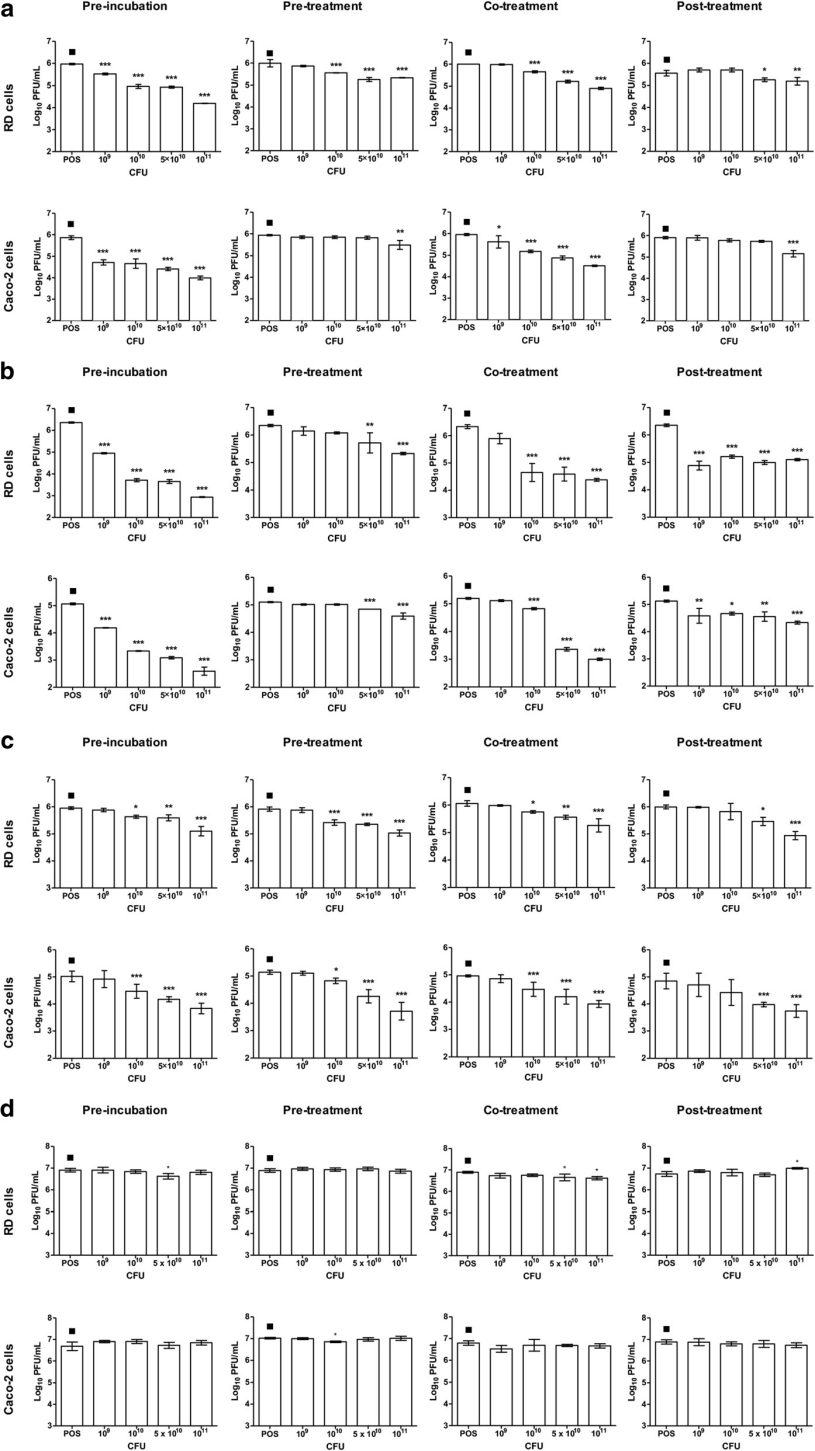
Antiviral effect of *L. reuteri* Protectis. Pre-incubation, pre-treatment, co-treatment and post-treatment setups were performed as detailed in Fig. 1. Virus titers in the supernatant of CA6- **a**, CA16- **b**, EV71- **c** and CB2- **d** infected RD cells and Caco-2 cells were determined by standard plaque assay in RD cells. A one-way ANOVA test with Dunnett’s posttest was performed (* *p <* 0.05, ** *p <* 0.005, *** *p <* 0.0005). Data are expressed as the mean ± standard deviation of technical triplicates. Two biological repeats were conducted. One representative is shown

Prior to evaluating the antiviral activity of *L. reuteri* Protectis bacteria in human RD and intestinal Caco-2 cell lines, a cell viability assay was performed to assess the cytotoxicity of these probiotic bacteria. Results indicated that 1 h incubation of up to 10^11^ CFU of live *L. reuteri* Protectis bacteria with RD and Caco-2 cell monolayers did not lead to significant cell viability loss (≥80 % viability) (Fig. 1). Next, various incubation conditions were performed to test the antiviral activity of *L. reuteri* Protectis (Fig. 2). In the pre-incubation set-up, bacteria and virus particles were incubated together for 1 h prior to infection of mammalian cells with the mixture. In the pre-treatment set-up, cell monolayers were incubated with bacteria for 1 h, and washed with PBS prior to virus infection. In the co-treatment set-up, cell monolayers were incubated for 1 h with virus and bacteria concomitantly. Finally, in the post-treatment set-up, cell monolayers were incubated with the virus for 1 h, and washed with PBS prior to incubation with bacteria for another hour. The culture supernatants were sampled at 12 or 24 h post-infection to determine the virus titers. CA6, CA16, CB2 and EV71 strains were tested. Virus alone, treated under the same experimental conditions, was used as a positive control (POS). The results indicated that *L. reuteri* Protectis bacteria displayed a significant antiviral activity against CA6, CA16 and EV71 but not against CB2 virus (Fig. 3a-d). Furthermore, the pre-incubation set-up where bacteria and virus are co-incubated prior to incubation with mammalian cells, led to the strongest reduction in virus titers compared to the positive control. This observation suggested a direct interaction between bacteria and virus particles, which may impair virus entry. The inhibitory effects observed were dose-dependent and virus-dependent whereby the greatest antiviral activity was observed against CA16 with more than 2–3 log reduction in virus titers when pre-mixed with 10^11^ bacteria (Fig. 3b). In the same conditions, reduction in CA6 and EV71 virus titers was approximately 2 log and 1 log, respectively (Fig. 3a and c). A dose-dependent antiviral activity was also observed in the co-treatment set-up where virus and bacteria were added concomitantly to the cell monolayers. However, reduction in virus titers was less dramatic than those obtained in the pre-incubation set-up. In contrast, no dose-dependent antiviral activity was clearly observed in the pre-treatment and post-treatment set-ups with CA6 and CA16 (Fig. 3a and b), but was seen with EV71 (Fig. 3c). Furthermore, comparable observations were made with both cell lines, suggesting that the antiviral activity of *L. reuteri* Protectis is not cell type-dependent and likely affects an important step of the infection cycle.

### Coxsackievirus type A and EV71 interact with live *L. reuteri* Protectis bacteria

**Fig. 4:**
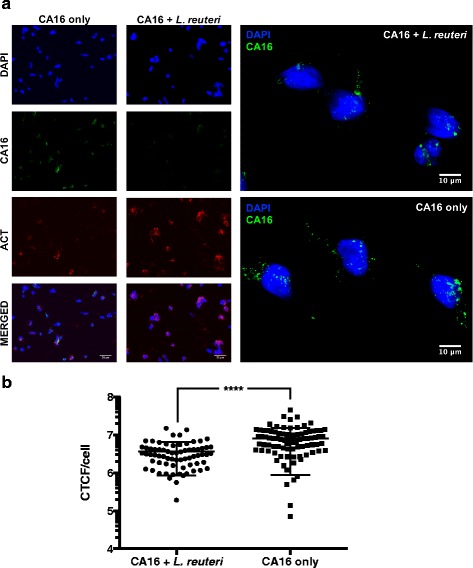
Immunostaining of RD cells infected with (*L. reuteri* + CA16) mixture. **a** *L. reuteri* Protectis bacteria (10^11^ CFU) were co-incubated with CA16 (10^5^ PFU) for 1 h in 37 °C prior to adding the mixture onto RD cells (10^5^ cells) for another 1 h. A control with RD cells infected with CA16 only was also performed. The monolayers were then washed thrice before methanol fixation and immunostained with anti-CA16 and anti-beta actin antibodies. Nuclei were also stained with DAPI. Images were taken using Olympus IX81 microscope. **b** Corrected total cell fluorescence (CTCF) (viral signal) was calculated from each cell originated from three random microscopic views. Statistical analysis was performed using Mann–Whitney *U* test (^****^, *p* < 0.0001)

The data obtained indicated that the pre-incubation set-up led to the greatest reduction in virus titers, thus suggesting that *L. reuteri* Protectis bacteria interfere with the virus entry step. To further test this hypothesis, *L. reuteri* bacteria and CA16 virus were co-incubated for 1 h in a cell free environment prior to infection of RD cells as described for the pre-incubation experimental setup. After 1 h infection, the cell monolayer was washed thoroughly, fixed and processed for immunostaining using anti-CA16 and anti-actin antibodies, while nuclei were stained with DAPI. The corrected total cell fluorescence (CTCF) specific to the virus signal (green) was calculated and indicated significantly lower signal intensity with the (CA16 + *L. reuteri*)-infected cells compared to CA16-infected cells only (Fig. 4). These data therefore further supported that *L. reuteri* bacteria interfere with CA16 entry into mammalian cells.

**Fig. 5:**
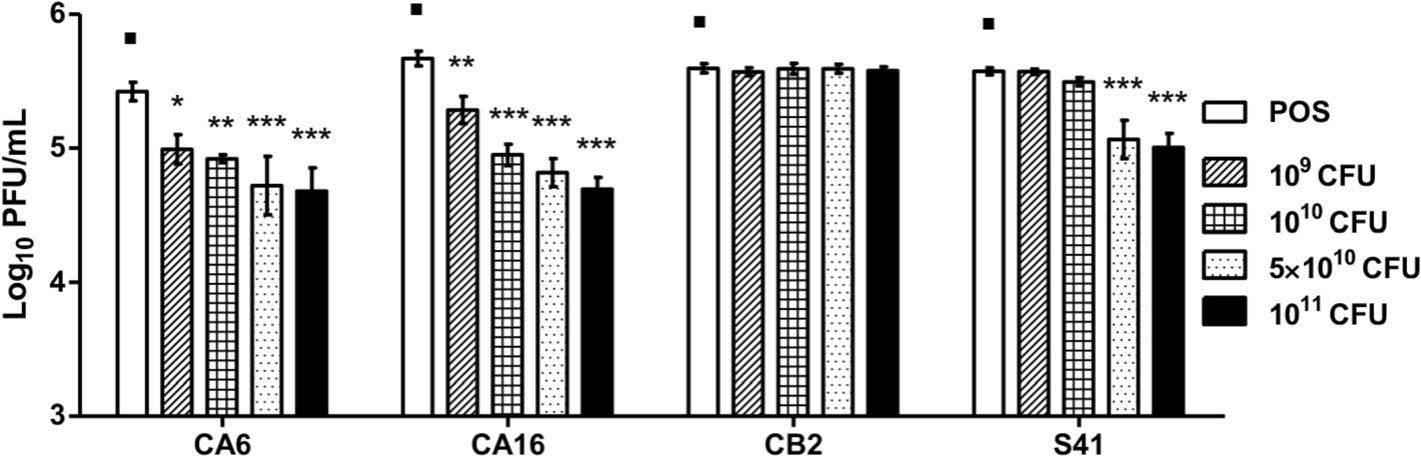
Virus-bacteria binding assay. Different quantities of live *L. reuteri* Protectis bacteria were incubated with a fixed amount of EV71, CA6, CA16 or CB2 virus particles for 1 h. The mixtures were then centrifuged and filtered to obtain free virus in the supernatant. Virus titers were determined by plaque assay using RD cells. A one-way ANOVA test with Dunnett’s posttest was performed (* *p <* 0.05, ** *p <* 0.005, *** *p <* 0.0005). Data are expressed as the mean ± standard deviation of technical triplicates. Two biological repeats were conducted. One representative is shown

We next asked whether *L. reuteri* bacteria physically interact with the virus particles during the pre-incubation phase, thereby compromising viral entry into the mammalian cells subsequently. To test this hypothesis, a dose range of live *L. reuteri* Protectis bacteria were co-incubated with a fixed amount of virus particles for one hour in a mammalian cell-free system. The mixtures were then centrifuged, the supernatants were collected and filtered to remove intact bacteria, and the amount of virus particles was determined by plaque assay. The results indicated a reduction in concentration of virus particles in the supernatant compared to the virus alone control (Fig. 5). This reduction was dependent on the amount of bacteria that were co-incubated with the virus and was seen with CA6, CA16 and EV71, but not with CB2 virus, thus correlating with the observation that *L. reuteri* Protectis bacteria do not impact CB2 infectivity (Fig. 3). Together, these data support that *L. reuteri* Protectis bacteria interact directly and physically with CA6, CA16 and EV71 virus particles and likely interfere with viral entry into the mammalian cells.

### Dead intact *L. reuteri* Protectis inhibits coxsackievirus type A infection

**Fig. 6:**
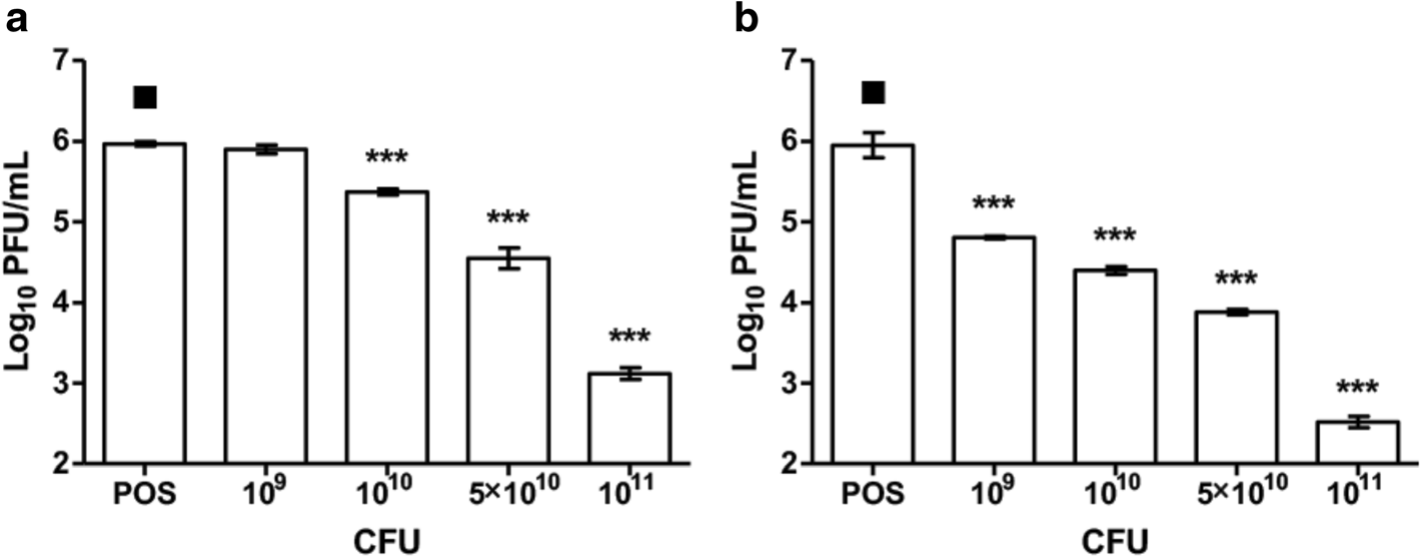
Antiviral effect of formaldehyde-inactivated *L. reuteri* Protectis. Formalin-fixed *L. reuteri* Protectis bacteria were bacteria were pre-incubated with CA6 or CA16 virus according to the pre-incubation set-up with Caco-2 cells. Virus titers in the supernatant of CA6- (**a**) and CA16- (**b**) infected cells were determined by standard plaque assay in RD cells. A one-way ANOVA test with Dunnett’s posttest was performed (* *p <* 0.05, ** *p <* 0.005, *** *p <* 0.0005). Data are expressed as the mean ± standard deviation of technical triplicates. Two biological repeats were conducted. One representative is shown

We next asked whether live *L. reuteri* Protectis bacteria were necessary to display a significant antiviral activity. To test this hypothesis, formaldehyde-treated *L. reuteri* Protectis bacteria were pre-incubated with CA6 or CA16 virus according to the pre-incubation set-up described above. The data showed that a bacteria dose-dependent reduction in virus titers was observed that was comparable to that observed with live *L. reuteri* Protectis bacteria (Fig. 6). Therefore, the data indicated that the antiviral activity of *L. reuteri* Protectis bacteria does not depend on bacteria replication and/or bacterial product secretion. They support that the antiviral mechanism relies on a physical interaction between bacteria and virus particles which likely interferes with the ability of the virus to bind to its mammalian receptor(s).

### *L. casei* Shirota bacteria do not display significant antiviral activity against CA16 and EV71

**Fig. 7:**
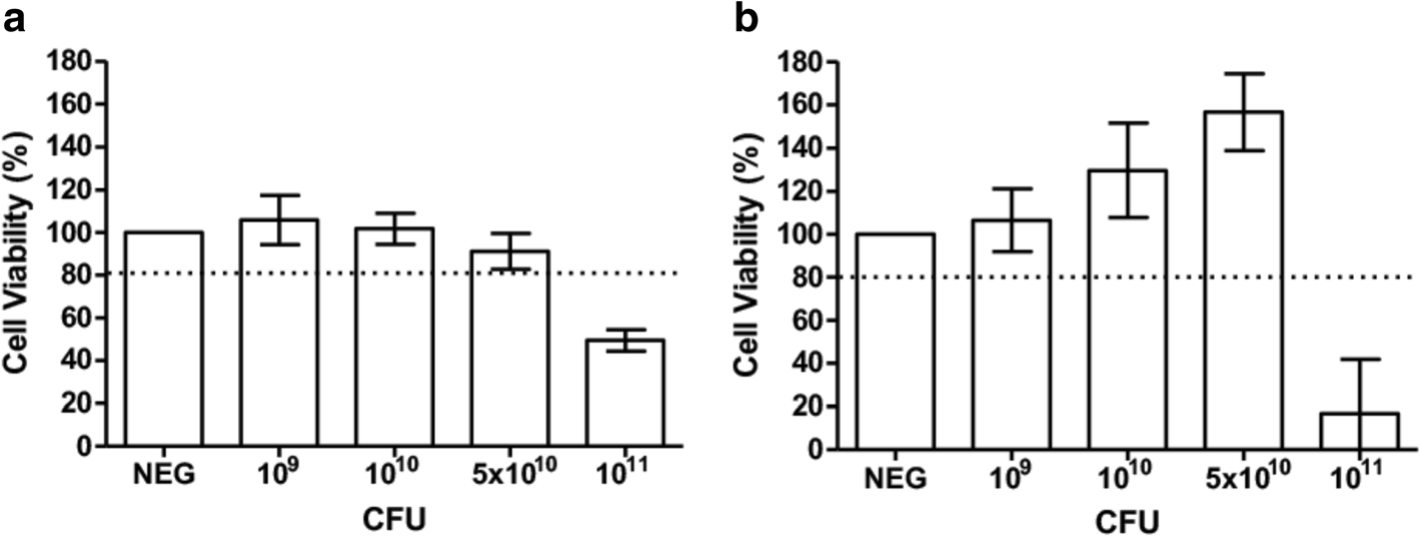
Cell viability in the presence of live *L. casei* Shirota. Different concentrations of bacteria were added to RD (**a**) and Caco-2 (**b**) cells as indicated and incubated for 1 h, then washed with 1xPBS thrice before 50 μg/ml gentamicin-supplemented maintenance media was added to the cells. Alamar blue assay was performed at 24 h post-treatment according to the manufacturer’s instructions. Data are expressed as the mean ± standard deviation of technical triplicates. Two biological repeats were conducted. One representative is shown

The potential antiviral activity of another widely consumed probiotic bacterium namely *L. casei* Shirota was explored. First, the cytotoxicity of *L. casei* Shirota was determined by incubating a dose range of bacteria with RD and Caco-2 cells. The results indicated that 10^11^ CFU of live *L. casei* Shirota bacteria appears to be toxic (< 80 % viability) to both RD cells and Caco-2 cells (Fig. 7). This cytotoxicity is likely due to the high amounts of lactic acid produced by *L. casei* Shirota bacteria which results in acidification of the culture medium as evidenced by the colour shift of the pH indicator (data not shown). Therefore, the antiviral assays were conducted with concentrations of *L. casei* Shirota ranging from 10^9^ to 5 × 10^10^ CFU.

**Fig. 8:**
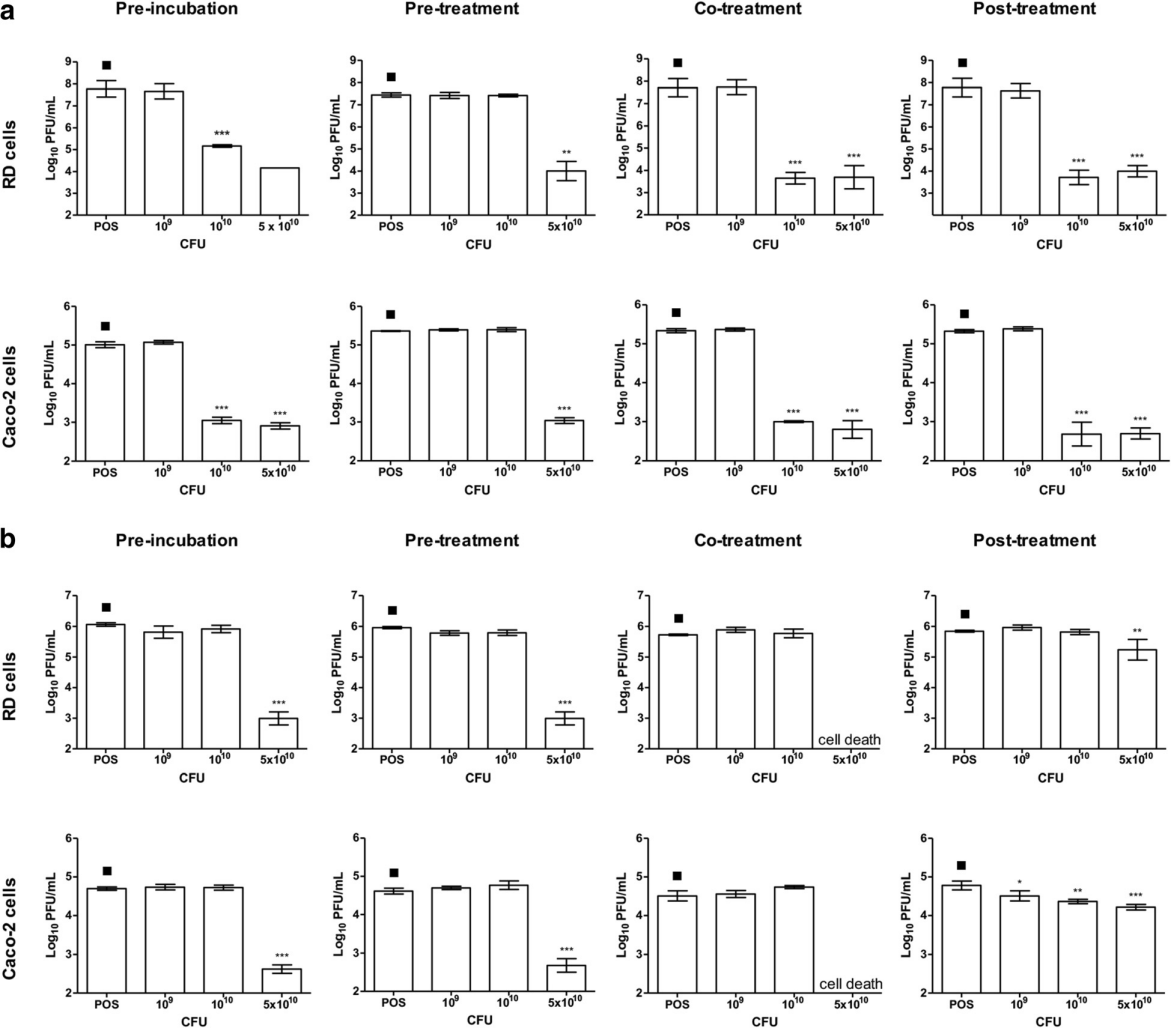
Antiviral effect of *L. casei* Shirota. Pre-incubation, pre-treatment, co-treatment and post-treatment setups were performed as detailed in Fig. 1. Virus titers in the supernatant of CA16- (**a**) and EV71- (**b**) infected RD cells and Caco-2 cells were determined by standard plaque assay in RD cells. A one-way ANOVA test with Dunnett’s posttest was performed (* *p <* 0.05, ** *p <* 0.005, *** *p <* 0.0005). Data are expressed as the mean ± standard deviation of technical triplicates. Two biological repeats were conducted. One representative is shown

The antiviral activity against CA16 and EV71 of *L. casei* Shirota was assayed in the pre-incubation, pre-treatment, co-treatment and post-treatment set-ups. The results indicated significantly lower CA16 virus titers with *L. casei* Shirota concentrations of 10^10^ and/or 5 × 10^10^ CFU in all the experimental set-ups (Fig. 8a). However, significant cytotoxicity was clearly observed at these bacterial concentrations, with cells lifting off from the bottom of the wells (data not shown). Similar observations were made with EV71-infected cells (Fig. 8b). Therefore, these data indicate that *L. casei* Shirota does not appear to display a significant antiviral activity against CA16 and EV71.

## Discussion

Despite increasing interests from the scientific community, development of effective prophylactic and therapeutic strategies against HFMD remains overdue. Thanks to their high safety profile, probiotics have been reported as an alternative preventive and therapeutic approach to treat a number of illnesses in infants and young children, in particular those affecting the GI tract [39].

Upon ingestion, coxsackieviruses and EV71 establish infection at the gastric mucosa, the primary site of infection, from where the viral particles transiently disseminate systemically and accumulate in muscles where the virus multiplies extensively [7, 40]. Subsequently, EV71 is believed to gain access to the CNS at the neuromuscular junctions and migrate to the brainstem via retrograde axonal transport [40, 41]. Based on this model of infection, the antiviral effect of probiotic bacteria against coxsackieviruses and EV71 was tested in vitro using relevant cell lines namely human skeletal muscle RD and intestinal Caco-2 cells. Our data clearly demonstrate a significant antiviral activity of *L. reuteri* Protectis against CA16, CA6 and EV71 but not CB2 virus. In contrast, *L. casei* Shirota did not display any significant antiviral effect against CA16 or EV71, indicating that the antiviral effect observed with *L. reuteri* Protectis is specific and limited to this probiotic bacterium.

*L. reuteri* is a commensal bacterium that can be found in the gut flora of some mammals and birds. Administration of *L. reuteri* to human babies, children and adults (including HIV patients) is safe and has been used for more than 10 years as probiotics. *L. reuteri* was shown in a number of clinical trials to enhance protection against a variety of diseases of microbial (including rotavirus, *Gardnerella vaginalis*, and *Helicobacter pylori* infection), chemical and environmental origin. In addition to maintaining the balance among the GI microbiota, which is the primary role of probiotics, *L. reuteri* has been reported to display some immuno-modulatory properties through the modulation of inflammatory cytokines and chemokines production by enterocytes and immunocytes, thereby influencing the host mucosal immune responses [22, 23]. Furthermore, studies have shown that reuterin secreted by *L. reuteri* has antimicrobial properties against Gram positive and Gram negative bacteria, as well as yeast, moulds and protozoa [42, 43]. The mode of action of reuterin remains speculative although inhibition of DNA synthesis and induction of oxidative stress in the target microorganisms have been proposed. A few studies have reported the antiviral effect of other probiotics through the production of antimicrobial molecules such as bacteriocins [39] or cell wall components [44]. However, in our study, two main lines of experimental evidence support that the antiviral effect of *L. reuteri* Protectis against CA and EV71 is unlikely to be mediated by the production of a soluble antimicrobial molecule such as reuterin. Firstly, filtered culture supernatant from *L. reuteri* Protectis harvested during the exponential or stationary phase, failed to show antiviral effect in the various experimental set-ups (data not shown). Secondly, dead bacteria (formalin-fixed) retain their antiviral property. Furthermore, pre-incubation of *L. reuteri* Protectis bacteria with CA or EV71 showed a significant dose-dependent reduction of virus titers which suggests a physical interaction between bacteria and viral particles that may interfere with virus entry into the mammalian host cell. This hypothesis is further supported by the observation of reduced virus titers in the supernatant of *L. reuteri* Protectis-virus mixtures after centrifugation and filtration. In addition to a direct binding of bacteria to the viral particles that likely interferes with the entry step (pre-incubation set-up), competition for attachment sites on cell surface between bacteria and virus (antiviral activity observed in co-treatment and post-treatment set-ups) could also contribute to the antiviral effect observed. Further work is necessary to elucidate the mechanisms by which *L. reuteri* Protectis interacts physically with CA16, CA6 and EV71.

The next logical step would be to test the antiviral activity of *L. reuteri* Protectis in animal models of CA and EV71 infection. So far mouse models of HFMD have employed the intraperitoneal, intramuscular or intracranial routes to establish infection [45, 46]. These routes of infection are not suitable to test the antiviral efficacy of *L. reuteri* Protectis which likely relies on a direct and local interaction between bacteria and the virus particles in the GI-tract. The oral route of infection in these animal models has proven less successful due to the existence of specific oral bottlenecks represented by physical barriers (colonic epithelium) that limit virus trafficking from the gut to other body sites [47]. Alternatively, EV71 oral infection of non-human primates has been reported [46, 47] and could be employed to test the antiviral effect of *L. reuteri* Protectis. However, economic and ethical aspects must be carefully considered.

## Conclusion

In conclusion, our work indicates a significant antiviral activity of *L. reuteri* Protectis against the main agents responsible for HFMD. Should these in vitro findings be translated in vivo, they would strongly suggest that *L. reuteri* Protectis has the potential to significantly impact positively on HFMD epidemics. However, due to the lack of a suitable in vivo model, and owing to the excellent safety profile of this probiotic in babies and young children, direct translation of this pre-clinical work to human application may be possible and could be quickly implemented in relevant communities. Such probiotics-based therapeutic approach may address the urgent need for a safe and effective means to protect against HFMD and limit its transmission among children.

## Abbreviations

ANOVA, one-way analysis of variance; CA, coxsackievirus type A; CB2, coxsackievirus type B strain 2; CFU/mL, colony-forming unit per mL; DMEM, Dulbecco’s modified eagle medium; EV71, enterovirus 71; FBS, fetal bovine serum; GI, gastrointestinal; HFMD, hand, foot and mouth diseases; *L. casei* Shirota, *Lactobacillus casei* Shirota; *L. reuteri* Protectis, *Lactobacillus reuteri* Protectis; LAB, lactic acid bacteria; MOI, multiplicity of infection; *p*, probability values; PBS, phosphate buffered saline; PFU/mL, plaque-forming unit per mL; POS, positive control; RD, rhabdomyosarcoma; SD, standard deviation
