# Erratum to: Antiviral activity of *Lactobacillus reuteri* Protectis against Coxsackievirus A and Enterovirus 71 infection in human skeletal muscle and colon cell lines

**DOI:** 10.1186/s12985-016-0633-0

**Published:** 2016-11-17

**Authors:** Lei Yin Emily Ang, Horng Khit Issac Too, Eng Lee Tan, Tak-Kwong Vincent Chow, Lynette Pei-Chi Shek, Elizabeth Huiwen Tham, Sylvie Alonso

**Affiliations:** 1 Department of Microbiology and Immunology, Yong Loo Lin School of Medicine, National University of Singapore, Centre for Life Sciences, 28 Medical Drive, #03-05, Singapore, 117456 Singapore; 2 Immunology programme, Life Sciences Institute, National University of Singapore, Singapore, Singapore; 3 Department of Paediatrics, National University Hospital, Singapore, Singapore; 4 Centre for Biomedical & Life Sciences, Singapore Polytechnic, Singapore, Singapore

## Erratum

Upon publication, the authors noticed that in the original version of the article [1], two of the authors’ names, ‘Lynette Pei-Chi Shek’ and ‘Elizabeth Huiwen Tham’ in this article do not appear correctly on our website and therefore on the PubMed citation.

The two authors appear on our website as 'Pei-Chi Lynette Shek' and 'Elizabeth Tham', when they should be 'Lynette Pei-Chi Shek' and 'Elizabeth Huiwen Tham' respectively.

This means that on PubMed they are currently “Tham E” and “Shek PC”. They should instead appear as “Tham EH” and “Shek LP” respectively.

We, the publishers, apologise for the incorrect way in which the names were displayed and for any inconvenience caused by this. This has now been corrected in this erratum.
